# Study on the Mechanism of Astragalus Polysaccharide in Treating Pulmonary Fibrosis Based on “Drug-Target-Pathway” Network

**DOI:** 10.3389/fphar.2022.865065

**Published:** 2022-03-08

**Authors:** Pingping Bing, Wenhu Zhou, Songwen Tan

**Affiliations:** Academician Workstation, Changsha Medical University, Changsha, China

**Keywords:** Astragalus polysaccharides, pulmonary fibrosis, network pharmacology, molecular docking, mechanism

## Abstract

Pulmonary fibrosis is a chronic, progressive and irreversible heterogeneous disease of pulmonary interstitial tissue. Its incidence is increasing year by year in the world, and it will be further increased due to the pandemic of COVID-19. However, at present, there is no safe and effective treatment for this disease, so it is very meaningful to find drugs with high efficiency and less adverse reactions. The natural astragalus polysaccharide has the pharmacological effect of anti-pulmonary fibrosis with little toxic and side effects. At present, the mechanism of anti-pulmonary fibrosis of astragalus polysaccharide is not clear. Based on the network pharmacology and molecular docking method, this study analyzes the mechanism of Astragalus polysaccharides in treating pulmonary fibrosis, which provides a theoretical basis for its further clinical application. The active components of Astragalus polysaccharides were screened out by Swisstarget database, and the related targets of pulmonary fibrosis were screened out by GeneCards database. Protein-protein interaction network analysis and molecular docking were carried out to verify the docking affinity of active ingredients. At present, through screening, we have obtained 92 potential targets of Astragalus polysaccharides for treating pulmonary fibrosis, including 11 core targets. Astragalus polysaccharides has the characteristics of multi-targets and multi-pathways, and its mechanism of action may be through regulating the expression of VCAM1, RELA, CDK2, JUN, CDK1, HSP90AA1, NOS2, SOD1, CASP3, AHSA1, PTGER3 and other genes during the development of pulmonary fibrosis.

## Introduction

Pulmonary fibrosis is caused by many causes, and it is a chronic, progressive and irreversible heterogeneous disease of pulmonary interstitial tissue (Raghu et al., 2011). Including pulmonary fibrosis caused by secondary factors and idiopathic pulmonary fibrosis. The pathological features of pulmonary fibrosis are mainly the proliferation of lung fibroblasts and excessive deposition of extracellular matrix, which leads to the destruction of lung tissue structure and eventually leads to chronic respiratory failure (Datta et al., 2011; King et al., 2011). There are many factors leading to pulmonary fibrosis, mainly including smoking and environmental exposure, genetic factors, virus infection and complications (Renzoni et al., 2014; Zaman and Lee, 2018). The data show that the incidence of pulmonary fibrosis is increasing year by year in the world and is similar to that of liver cancer, gastric cancer and cervical cancer (Hutchinson et al., 2015). At present, with the global spread of Coronavirus Disease 2019 (COVID-19), there may be more patients with pulmonary fibrosis. COVID-19 can cause severe lung injury and may lead to pulmonary fibrosis. Previous data of coronavirus infection (SARS-CoV or MERS-CoV) and new data of COVID-19 pandemic indicate that severe fibrosis may occur after SARS-CoV-2 infection (George et al., 2020). Early diagnosis of pulmonary fibrosis is difficult and incurable, and the prognosis is very poor. The median survival time after diagnosis is 2–3 years, The 5-year survival rate is less than 30% (Vancheri and du Bois, 2013; Wuyts et al., 2020). At present, the main treatments for pulmonary fibrosis are drug therapy and lung transplantation. Lung transplantation is the most effective treatment at present, but it is limited by the supply of donor organs, economic situation and patients’ situation (Kistler et al., 2014). As for drug therapy, so far only Pirfenidone and Nidanib have been approved by FDA for the treatment of pulmonary fibrosis worldwide (Mora et al., 2017; Lederer and Martinez, 2018). These two anti-fibrosis drugs can improve patients’ lung function, but they still can’t cure pulmonary fibrosis and have obvious side effects, such as diarrhea and drug-induced liver injury, anorexia, vomiting and photosensitive rash, etc. (Richeldi et al., 2014; King et al., 2014). At present, the pathogenesis of pulmonary fibrosis is not clear, but the incidence of pulmonary fibrosis is on the rise, especially with the pandemic of COVID-19, the incidence of pulmonary fibrosis secondary to COVID-19 will further increase, and seriously affect the health of patients. Although many treatments have been used to treat pulmonary fibrosis, there is still no safe and effective treatment. Therefore, it is of great significance to find drugs with high efficiency and less adverse reactions to improve the quality of life and survival rate of patients with pulmonary fibrosis. A large number of clinical and experimental studies show that traditional Chinese medicine can obviously improve many pathological links of pulmonary fibrosis, and the toxicity of traditional Chinese medicine is relatively small, which can improve the quality of life of patients and reduce the mortality rate, and has a good application prospect (Zhao et al., 2017). Among them, Astragalus membranaceus plays an important role in treating fibrosis (Ma et al., 2020). Astragalus membranaceus has been used in Chinese medicine for nearly 2000 years and is one of the most popular Chinese medicines in the world (Shahrajabian et al., 2019). As one of the traditional Chinese medicines commonly used in clinic, Astragalus membranaceus can improve and treat various diseases, and has many pharmacological effects, such as antioxidation (Shahzad et al., 2016), immunomodulation (Block and Mead, 2003), antifibrosis (Zhou et al., 2016), antitumor (Zhou et al., 2018) and analgesia (Di Cesare Mannelli et al., 2017). The main bioactive components in Astragalus membranaceus include polysaccharides, saponins, flavonoids and alkaloids (Shahrajabian et al., 2019). Among them, Astragalus polysaccharide is the most abundant component in Astragalus membranaceus, which has a variety of pharmacological activities (Zheng et al., 2020). The main components of Astragalus polysaccharides include dextran, heteropolysaccharide, acidic polysaccharide, and neutral polysaccharide. The monosaccharide components of Astragalus polysaccharide include glucose, glucuronic acid, arabinose, rhamnose, fructose, mannose, galactose, galacturonic acid, fucose, etc (Wang et al., 2019). Studies have shown that Astragalus polysaccharide has anti-fibrosis pharmacological activity, and the main active ingredient of Astragalus membranaceus in anti-pulmonary fibrosis may be Astragalus polysaccharide. However, due to the complex components of Astragalus polysaccharide, the research on the anti-pulmonary fibrosis mechanism of Astragalus polysaccharide is relatively lacking at present, and the anti-pulmonary fibrosis mechanism of Astragalus polysaccharide is still unclear (Li et al., 2011).

Network pharmacology is a new discipline of multi-target drug molecular design by network construction and selecting specific signal Nodes. Emphasis is placed on the multi-channel regulation of signal pathway, improving the therapeutic effect of drugs and reducing the toxic and side effects, thus opening up new ways for drug development and utilization and saving the research and development cost of drugs (Zhang et al., 2019). Molecular docking is a computer simulation of the recognition process between two or more molecules, which involves spatial matching and energy matching between molecules. Molecular docking methods are widely used in drug design, screening and other fields (Morris and Lim-Wilby, 2008). Based on network pharmacology and molecular docking technology, this study preliminarily explored the mechanism of Astragalus polysaccharides in anti-pulmonary fibrosis, provided possible therapeutic drugs for the treatment of pulmonary fibrosis, and laid a theoretical foundation for the clinical application of Astragalus polysaccharides in anti-pulmonary fibrosis.

## Methods

### Screening of Key Active Components and Targets in Astragalus Polysaccharides

HERB (http://herb.ac.cn/) is a platform for pharmacology of Chinese herbal medicines. The database mainly includes the chemical substances, targets and drug target networks of Chinese herbal medicines (Ru et al., 2014). We searched the HERB database for related chemical components with “Astragalus membranaceus” as the key word, and finally screened the components of Astragalus membranaceus polysaccharides from the above-mentioned searched compounds by combining with references. Pubchem database (https://pubchem.ncbi.nlm.nih.gov/) is a biological activity database of small organic molecules supported by the National Institutes of Health and maintained by the National Biotechnology Information Center (Li et al., 2010). We get the SMILES numbers of the compound components through Pubchem database. SwissTargetPrediction database (www.swisstargetprediction.ch/) is a database for target prediction of more than 3,000 kinds of protein and more than 370,000 kinds of known active substances of different species (Zoete et al., 2016). We import the SMILES of the obtained compounds into Swiss Target Prediction database to predict the target of Astragalus polysaccharide.

### Screening of Disease Targets

Enter the keyword “Pulmonary fibrosis” in the human gene database GeneCards (//www.genecards.org), and collect the disease targets related to pulmonary fibrosis (Safran et al., 2002). Then, with the help of jvenn (http://www.bioinformatics.com.cn/static/others/jvenn/example.html), the active ingredient targets of Astragalus polysaccharides screened out are intersected with the targets related to pulmonary fibrosis, and finally, the potential targets of Astragalus polysaccharides for treating pulmonary fibrosis are determined.

### Network Construction

Through the software Cytoscape (v3.7.1), the screened active ingredients and the potential targets are visualized by constructing the network between the active ingredients and the potential targets (Lopes et al., 2010). And further construct the target topology network by using BisoGenet. STRING database is a database for searching protein-protein interactions, which contains a large number of PPI data verified by experiments and predicted by bioinformatics methods. In order to analyze the interaction between the potential target genes and proteins of Astragalus polysaccharide in anti-pulmonary fibrosis, a PPI(protein-protein interaction) network was built by STRING database (https://cn.string-db.org/), which was set as *Homo sapiens* and high confidence of 0.700, and the key targets were screened by visualization and network topology heterogeneity analysis in Cytoscape software.

### KEGG and GO Enrichment Analysis

In order to describe and annotate the functions of target genes and explore the signal pathways of their functions, we input the selected core targets into the DAVID database (//www.genecards.org) (Huang et al., 2009). The species is limited to human, and the enrichment analysis of GO and KEGG is carried out with *p* < 0.05 as the screening condition. The enrichment analysis of GO includes the enrichment analysis of biological process, BP), cellular component, CC) and molecular function (MF). Through enrichment analysis, the biological processes and potential signal pathways involved in the treatment of pulmonary fibrosis by Astragalus polysaccharides were screened out. In addition, the pathway enrichment analysis of core targets is carried out by ClueGO.

### Molecular Docking

Generally, we judge the binding degree of ligand and receptor by the level of energy. When the conformation of compound molecule and receptor is stable, the lower the energy, the greater the possibility of action. We use molecular docking technology to study the binding ability of the selected target protein to active compounds. We obtained the 3D structure of the target protein obtained by the above screening through PDB database. With the active ingredient of Astragalus polysaccharide as the ligand, the processed ligand was molecularly docked with the target protein in Autodock Vina 1.1.2 software (Trott and Olson, 2010). Finally, the docking results are visualized by PyMOL 2.3.4 software.

## Result

### Screening of Key Active Components and Targets in Astragalus Polysaccharides

**FIGURE 1:**
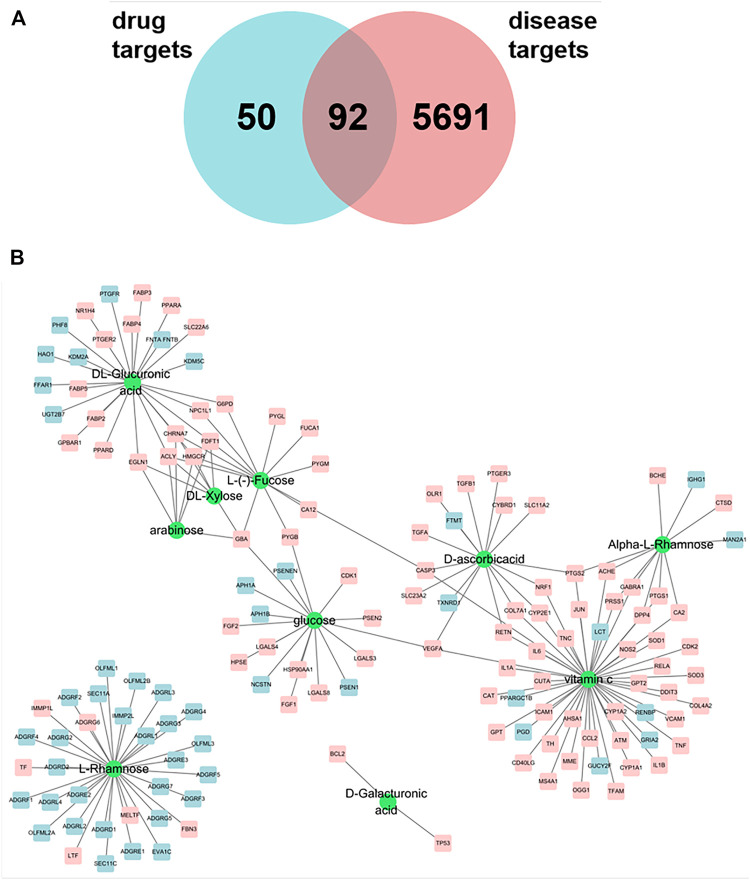
**(A)** Venn maps of components and disease targets have 92 common targets. **(B)** Components-Target network of astragalus polysaccharide. The green circle indicated the component, and the square was the action target of polysaccharide component, and the pink square was the potential treatment target of pulmonary fibrosis.

We searched the HERB database for the keyword “huang qi” to obtain the active ingredient chemistry of Astragalus membranaceus. Finally, we determined the ingredients of Astragalus membranaceus polysaccharide from these ingredients screened from Astragalus membranaceus. We obtained 10 kinds of polysaccharide ingredients, namely D-Galacturonic acid, Alpha-L-Rhamnose, D-ascorbicacid, Vitamin C, L-Rhamnose, DL-Glucuronic acid, L-(-)-Fucose, DL-Xylose, glucose, arabinose. Search the HERB database for related targets of Astragalus polysaccharides, and predict the targets of some polysaccharide components through Swiss Target Prediction database, and finally get 142 targets of Astragalus polysaccharides (Figure 1).

### Screening of Disease Targets

With “pulmonary fibrosis” as the key word, 5,783 genes were preliminarily screened out from Genecards database, and the potential targets of Astragalus polysaccharides for treating pulmonary fibrosis were obtained by intersecting the selected targets with disease targets by using jvenn (http://www.bioinformatics.com.cn/static/others/jvenn/example.html). As shown in Figure 1A, 142 related targets of Astragalus polysaccharides were screened, among which there were 92 potential targets for treating pulmonary fibrosis. We introduced ten compounds and corresponding targets in Astragalus polysaccharide into Cytoscape to construct a polysaccharide component-target network. As shown in Figure 1B, it can be seen from the network diagram that compounds act on multiple targets, and some targets also correspond to multiple compounds. Among them, the sub-network degree value of Vitamin C and related targets is high, and most targets are potential targets for treating pulmonary fibrosis, suggesting that Vitamin C may play an important role in the treatment of pulmonary fibrosis by Astragalus polysaccharides.

### KEGG and GO Enrichment Analysis

**FIGURE 2:**
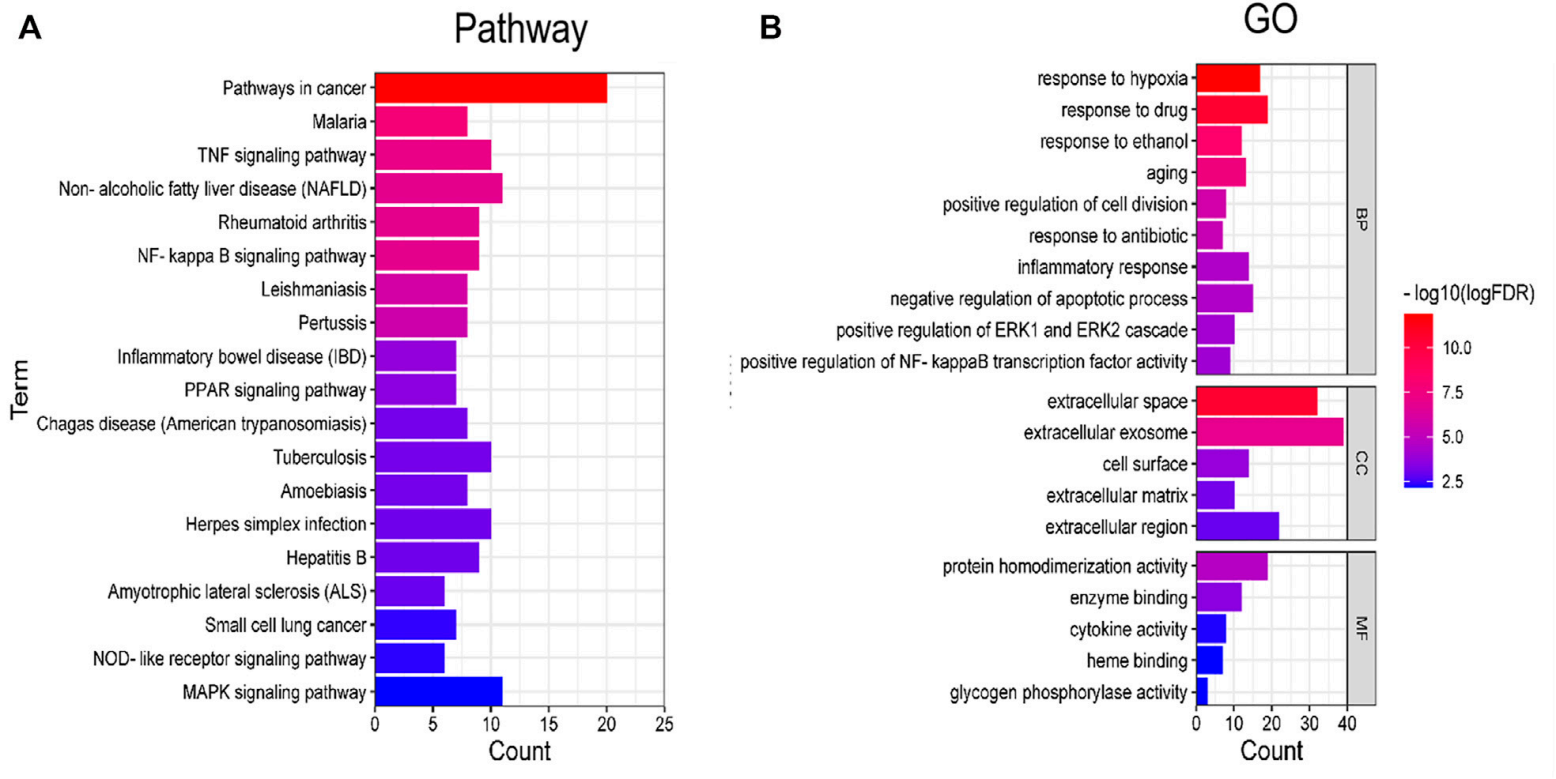
Enrichment analysis of KEGG and GO. **(A)** Pathway enrichment analysis. **(B)** GO enrichment analysis.

DAVID database was used for GO and KEGG enrichment analysis of Astragalus polysaccharide in treating pulmonary fibrosis. The results of KEGG pathway enrichment analysis are shown in Figure 2A, mainly including Pathways in cancer, Malaria, TNF signaling pathway, NF-kappa B signaling pathway, Rheumatoid arthritis, Non-alcoholic fatty liver disease (NAFLD), Leishmaniasis, Pertussis, Inflammatory bowel disease (IBD), PPAR signaling pathway, Tuberculosis, Chagas disease (American trypanosomiasis), Amoebiasis, Herpes simplex infection, Hepatitis B, Amyotrophic lateral sclerosis (ALS), Small cell lung cancer, NOD-like receptor signaling pathway, MAPK signaling pathway etc.

GO enrichment analysis results show that it mainly involves response to hypoxia, response to drug, response to ethanol, aging, positive regulation of cell division, response to antibiotic, inflammatory response, negative regulation of apoptotic process, positive regulation of ERK1 and ERK2 cascade, positive regulation of NF-kappaB transcription factor activity, extracellular space, extracellular exosome, cell surface, extracellular matrix, extracellular region, protein homodimerization activity, enzyme binding, cytokine activity, glycogen phosphorylase activity, heme binding etc. (Figure 2B).

### Network Construction and Screening of Core Targets

**FIGURE 3:**
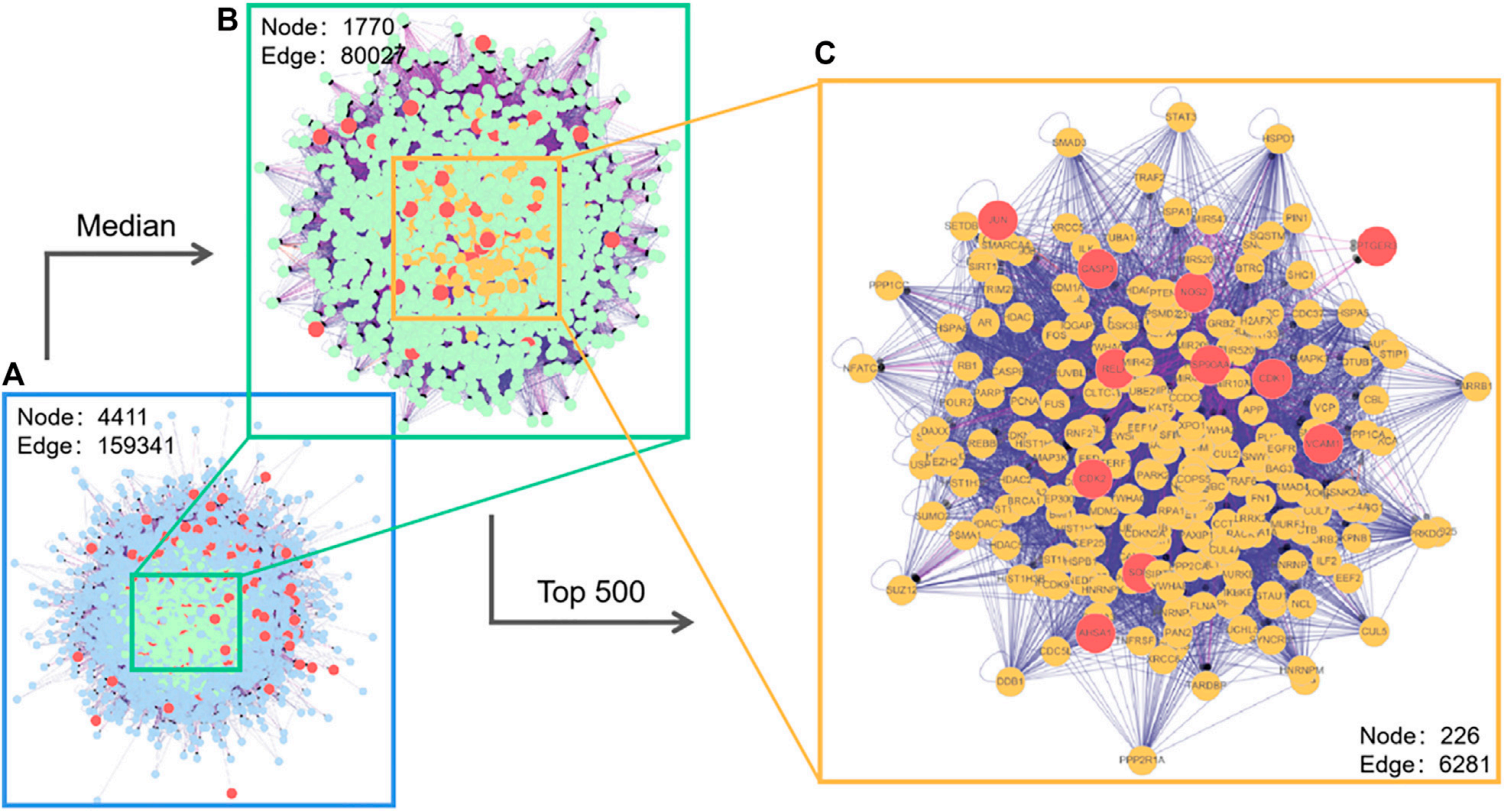
Interaction network of Astragalus polysaccharide targets (with magnified median figure and the top 500 points).

The protein interaction network of potential targets of Astragalus polysaccharide in treating pulmonary fibrosis was obtained by using the BisoGenet plug-in in Cytoscape. As shown in Figure 3, there are 4,411 node in network a and 159,341 target interaction relationships. In order to screen out the core targets of the network more accurately, network b was extracted with the median values of the three evaluation indexes of Betweenness, Degree and Closeness as the boundaries, and there were 1770 nodes and 80,027 kinds of target interactions in network b. We continued to extract the targets of the top 500 indexes in network b to build interactive network c, which had 226 nodes and 6,281 kinds of target interactions. In the core network c, we found 11 direct targets, VCAM1, RELA, CDK2, JUN, CDK1, HSP90AA1, NOS2, SOD1, CASP3, AHSA1, PTGER3, which had the greatest correlation with Astragalus polysaccharides in treating pulmonary fibrosis. Therefore, we regard these 11 targets as the potential core targets of Astragalus polysaccharides in treating pulmonary fibrosis.

### Construction and Enrichment Analysis of Core Target Network

**FIGURE 4:**
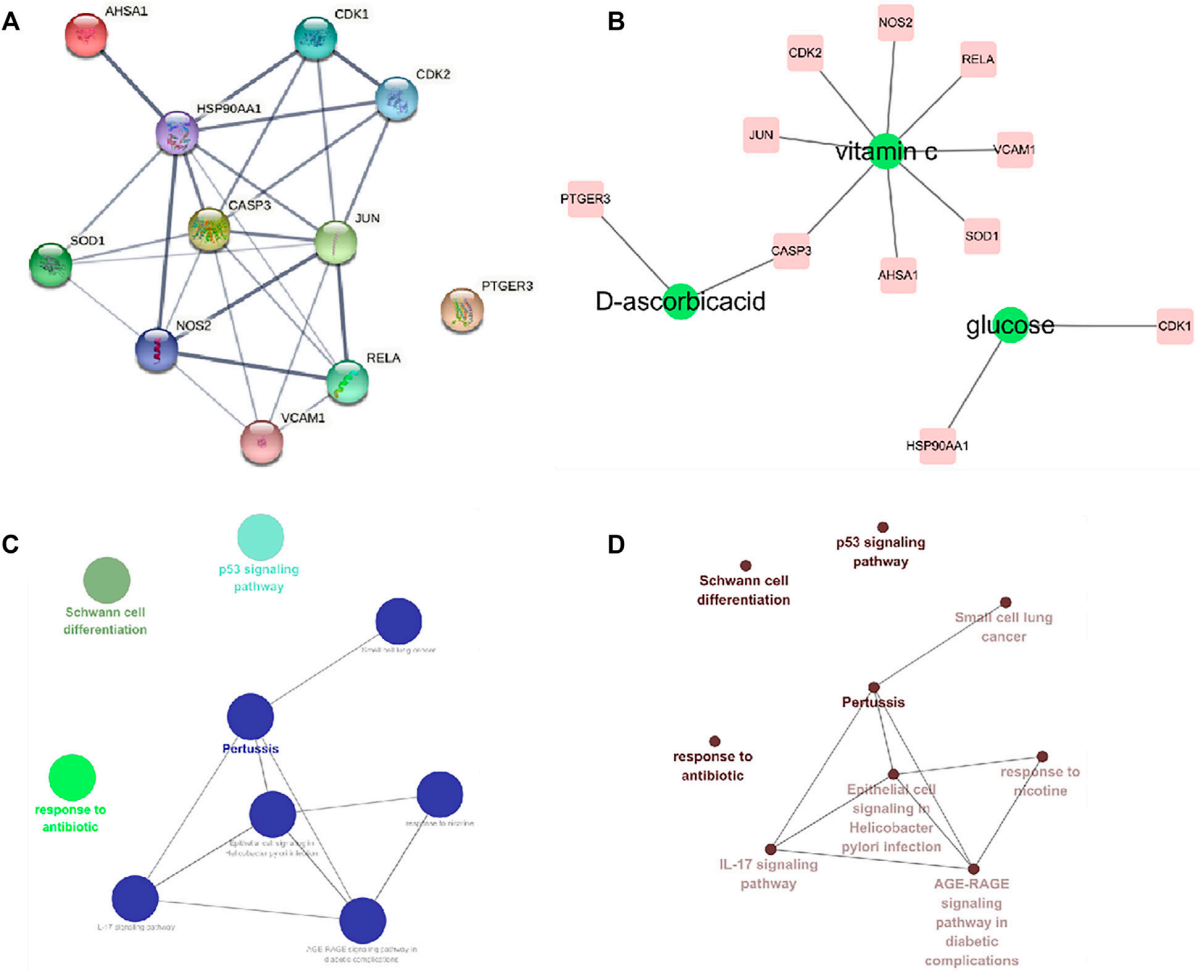
Interaction network of core targets and pathway enrichment analysis. **(A)** Interaction network of core targets. **(B)** Core target component network **(C,D)** Core targets pathway enrichment network.

We have further constructed PPI network for these 11 core targets, and found that JUN, CASP3 and HSP90AA1 have higher degrees in the network, suggesting that they may play a key role in the treatment (Figure 4A). In addition, we also screened out three components of Astragalus polysaccharides that act on the 11 core targets (D-ascorbicacid, Vitamin C, Glucose) (Figure 4B). And ClueGO was used to construct pathways enrichment network for the core targets, and the pathways enrichment includes Schwann cell differentiation, p53 signaling pathway, response to antibiotic, Pertussis, Small cell lung cancer, IL-17 signaling pathway, Epithelial cell signaling in *Helicobacter pylori* infection, response to nicotine, AGE-RAGE signaling pathway in diabetic complications. Among them, four types were obtained by clustering (Figures 4C,D).

### Molecular Docking

**FIGURE 5:**
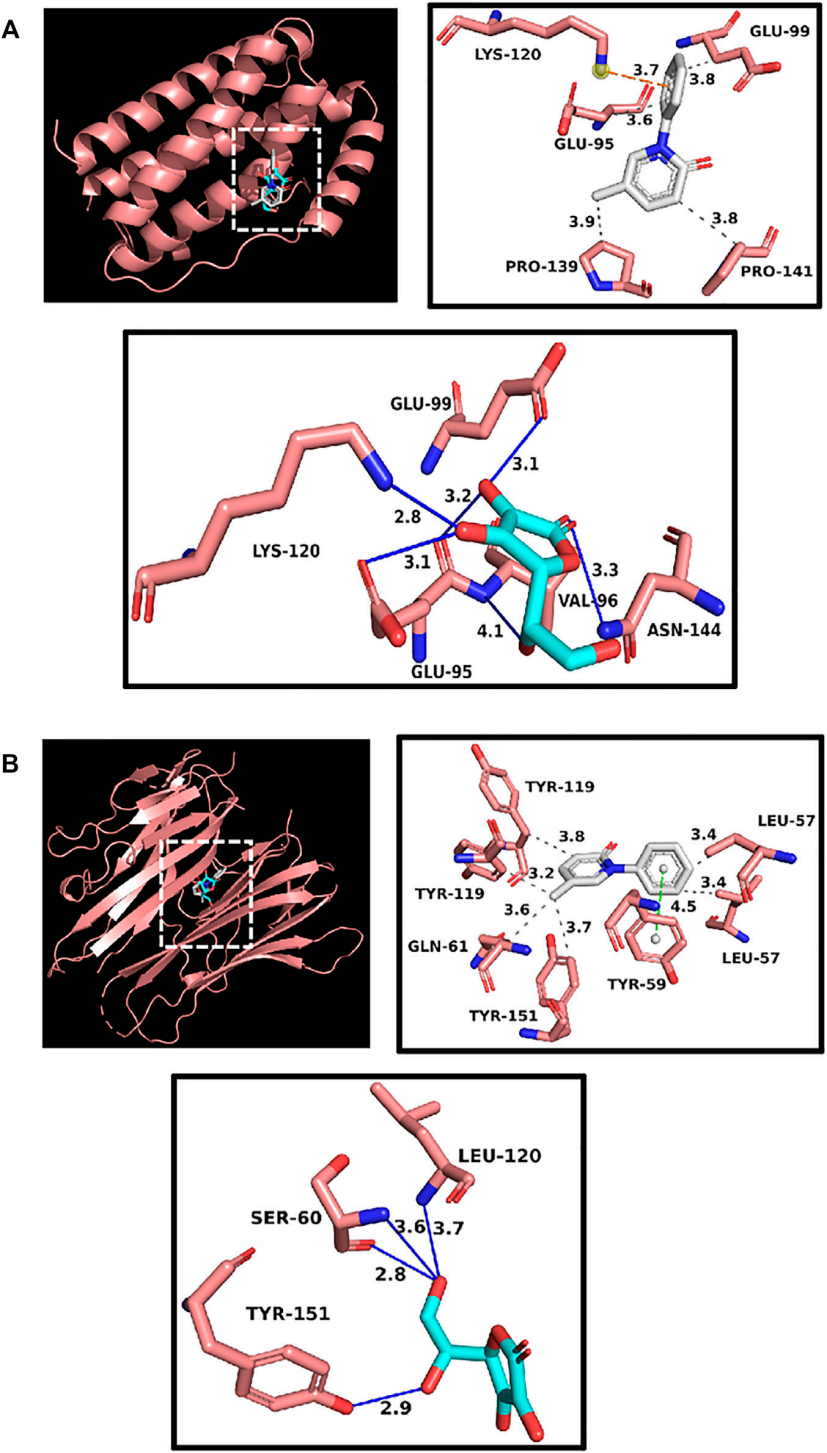
**(A)** Docking display of pirfenidone and Vitamin C with IL6 respectively. Gray compound represents pirfenidone, and blue compounds represent Vitamin C. **(B)** Docking display of pirfenidone and Vitamin C with TNFα respectively. Gray compound represents pirfenidone, and blue compounds represent Vitamin C.

**FIGURE 6:**
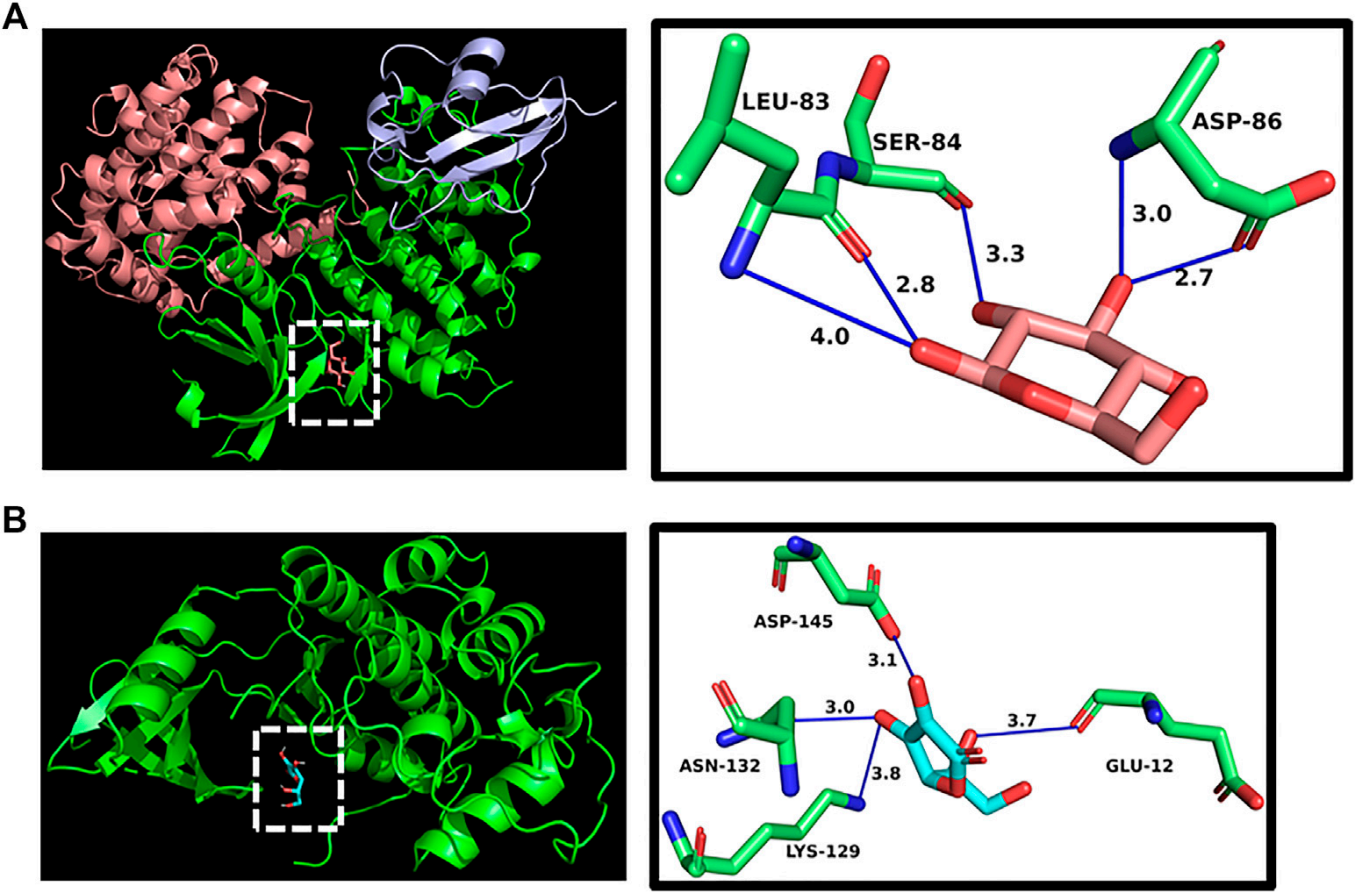
**(A)** Docking display of CDK1 and glucose. **(B)** Docking display of CDK2 and Vitamin C.

Up to now, only pirfenidone and Nidanib have been approved by FDA to treat pulmonary fibrosis in the world, and these two drugs have a good clinical effect on pulmonary fibrosis. According to previous reports, pirfenidone can inhibit targets such as IL6 and TNFα, and through the previous component target network construction, we found the polysaccharide component (Vitamin C) that acts on these two targets, so we follow up with the docking scores of pirfenidone with IL6 and TNFα as reference values. The results show that the affinity scores of Vitamin C, an active compound in Astragalus polysaccharides, with these two targets are similar to those of pirfenidone, indicating that the key active substances in Astragalus polysaccharides may play an anti-pulmonary fibrosis role, and the molecular docking is shown in Figure 5. In addition, we have docked two core targets, CDK1 and CDK2, and docked the Glucose and Vitamin C acting on these two targets into the active pockets of small molecule inhibitors of CDK1 and CDK2, respectively. CDK1 and CDK2 belong to cyclin-dependent protein kinases, which are important factors in cell cycle regulation. It has been reported that lung fibrosis can be controlled to some extent by inhibiting CDKs. It was found that the affinity was similar to the docking scores of IL6 and TNFα, indicating that these key active components may play a key role in the treatment of pulmonary fibrosis (Figure 6).

## Discussion

For a long time, the treatment of pulmonary fibrosis has been a difficult problem in the world. Except for lung transplantation, there is still a lack of very effective treatment drugs and methods (Kistler et al., 2014). At present, the western medicines used clinically to treat pulmonary fibrosis, such as pirfenidone and Nidanib, can only slow down the decline rate of pulmonary function in patients with mild to moderate pulmonary fibrosis, but the adverse reactions are obvious and expensive. The clinical mortality rate of pulmonary fibrosis is not lower than that of tumor, and the incidence rate is increasing year by year. Due to the pandemic of COVID-19, the incidence rate will further increase (Hutchinson et al., 2015; George et al., 2020). However, there is no ideal therapeutic drug at present, and the pathogenesis is not completely clear and complicated. Therefore, it is very meaningful to find more effective therapeutic targets and drug research and development. In recent years, with the deepening of research on pulmonary fibrosis, the exploration of treating pulmonary fibrosis with traditional Chinese medicine has gradually increased, and great progress has been made in treating pulmonary fibrosis with traditional Chinese medicine, which has attracted much attention because of its multi-channels, multi-targets, few side effects and remarkable curative effect (Ji et al., 2020; Yu et al., 2020). Astragalus polysaccharide is an important active substance in Astragalus membranaceus. Studies have shown that Astragalus polysaccharide has anti-fibrosis pharmacological activities (Li et al., 2011). However, the components of Astragalus polysaccharides are complex, and at present, there is a lack of research on the mechanism of Astragalus polysaccharides against pulmonary fibrosis, and the mechanism of Astragalus polysaccharides against pulmonary fibrosis is still unclear. In this study, the core pharmacodynamic substances and targets were preliminarily determined by network pharmacology, and the core targets were enriched by GO and KEGG pathways. Finally, the molecular docking study of these core active components and core targets was carried out by AutoDock Vina to verify the analysis results of network pharmacology. Ten kinds of active ingredients in Astragalus polysaccharides were screened out, among which Ascorbicacid, Glucose and D-Ascorbicacid are the core targets in the network, suggesting that these active ingredients play an important role in the treatment of pulmonary fibrosis by Astragalus polysaccharides. By analyzing the target interaction network VCAM1,RELA,CDK2,JUN,CDK1,HSP90AA1,NOS2, SOD1,CASP3,AHSA1, PTGER3 are at the core of the network, which can be regarded as the key targets of Astragalus polysaccharides in treating pulmonary fibrosis.

Marcia Rodrigues et al.’ s research shows that Asorbicacid can treat pulmonary fibrosis caused by paraquat poisoning, and the results of animal experiments show that Asorbic acid mainly treats pulmonary fibrosis by reducing the number of macrophages, neutrophils and lymphocytes in fibrotic mice, reducing the levels of IL-6, TGF-β and IL-17 and increasing the activity of antioxidant enzymes in lung homogenate (Rodrigues da Silva et al., 2018). It was suggested that glucose metabolism may be beneficial to prevent myofibroblast differentiation in idiopathic pulmonary fibrosis (Azuelos, 2020). VCAM1 is upregulated in idiopathic pulmonary fibrosis, which can induce TGF-β1, and its main mechanism is to inhibit the proliferation of fibroblasts by reducing G2/M and S phases of cell cycle (Agassandian et al., 2015). Previous studies have shown that the phosphorylation of RELA on Ser536 will affect hepatic myofibroblasts. For pulmonary fibrosis, current studies have shown that the phosphorylation of RELA on Ser536 may be the core regulator of pulmonary fibroblasts (Moles et al., 2013). JNK is a member of mitogen-related protein kinase family. Studies have shown that JNK plays an important role in pulmonary fibrosis, in which JNK1 promotes collagen deposition and leads to further development of pulmonary fibrosis (Alcorn et al., 2009).

Through GO enrichment analysis of the key targets of effective components of Astragalus membranaceus, it is confirmed that Astragalus membranaceus has certain influence on many biological processes, such as response to hypoxia, aging, positive regulation of cell division, response to antibiotic, inflammatory response, negative regulation of apoptotic process, etc. Pathways related to pulmonary fibrosis in KEGG pathway mainly include Pathways in cancer, Malaria, TNF signaling pathway, Non-alcoholic fatty liver disease (NAFLD), Rheumatoid arthritis, NF- kappa B signaling pathway, Pertussis, Tuberculosis, Herpes simplex infection, Hepatitis B, Small cell lung cancer etc.

The results of molecular docking show that the binding properties of the main active components of Astragalus polysaccharides to the key targets selected from the research results are similar to those of the control drug pirfenidone, which indicates that the key active components of Astragalus polysaccharides can stably bind to receptors and play an anti-pulmonary fibrosis role. The molecular docking prediction results provide further evidence for the active components of Astragalus polysaccharide to act on specific targets to treat pulmonary fibrosis.

In this study, a “compound-target” network was established by the method of network pharmacology, and the relationship between pulmonary fibrosis, active components of Astragalus polysaccharides and their targets was studied as a whole. The key compounds and core targets of Astragalus polysaccharides for treating lung cancer and their action pathways were screened, and the multi-component, multi-target and multi-pathway action mechanism of anti-lung cancer was preliminarily discussed, which provided important theoretical clues and basis for further in-depth study on the medicinal material basis and action mechanism of Astragalus polysaccharides for treating pulmonary fibrosis. In this paper, the mechanism of action of Astragalus polysaccharide is discussed based on its chemical components and targets, but it cannot fully reflect the influence of metabolites of Astragalus polysaccharide on the mechanism of action. Therefore, the results of this study still have some limitations, and it is necessary to further verify the results of pharmacological prediction analysis based on network.

## Data Availability

The original contributions presented in the study are included in the article/Supplementary Material, further inquiries can be directed to the corresponding authors.
